# Entomopathogenic fungi based microbial insecticides and their physiological and biochemical effects on *Spodoptera frugiperda* (J.E. Smith)

**DOI:** 10.3389/fcimb.2023.1254475

**Published:** 2023-12-11

**Authors:** Perumal Vivekanandhan, Kannan Swathy, Alford Lucy, Pittarate Sarayut, Krutmuang Patcharin

**Affiliations:** ^1^ Office of Research Administration, Chiang Mai University, Chiang Mai, Thailand; ^2^ Department of Entomology and Plant Pathology, Faculty of Agriculture, Chiang Mai University, Chiang Mai, Thailand; ^3^ School of Biological Sciences, Life Sciences Building, University of Bristol, Bristol, United Kingdom

**Keywords:** fungal insecticide, acetylcholinesterase, α-carboxylesterase, and β-carboxylesterase, natural microbial insecticide, target specific, enzyme response

## Abstract

**Background:**

‘The fall armyworm, *Spodoptera frugiperda*’, represents a significant threat to maize production, a major staple crop in Asian countries.

**Methods:**

In pursuit of more effective control of this insect pest, our study assessed the physiological and biochemical effects of the entomopathogenic fungus *Metarhizium anisopliae* against the larvae of *S. frugiperda*.

**Results:**

Results revealed that, following nine days of treatment, a high concentration of conidia (1.5x10^7^ conidia/mL^-1^) was toxic to all stages of larvae (second to fifth instar), resulting in 97% mortality of the second instar, 89% mortality of the third instar, 77% mortality of the fourth instar, and 72% mortality of fifth instar. All larval instars were found to have dose-dependent mortality effects. Treated *S. frugiperda* larvae further displayed significant physiological, morphological, and behavioral changes. Here, treated larvae displayed significantly lower levels of acetylcholinesterase, α-carboxylesterase, and β-carboxylesterase enzyme activity when compared to control groups. Treated larvae underwent an outward morphological change as the result of a decrease in the exterior cuticle of the anal papillae and a demelanization of the interior cuticle. Treated larvae also exhibited abnormal feeding behaviors as a consequence of the negative impact of conidia treatment on the neuromuscular system. Investigation into the effect of *M. anisopliae* on the non-target organism, the earthworm *Eudrilus eugeniae*, revealed that *M. anisopliae* conidia did not produce significant pathogenicity following three days of treatment. Furthermore, histological analysis revealed no significant effect of the entomopathogenic fungi on the gut tissue of the non-target organism.

**Conclusion:**

This study highlights the potential of *M. anisopliae* in the control of *S. frugiperda*.

## Highlights

• Highest larval mortality (97%) of *S. frugiperda* 2^nd^ instar larvae was obtained with a concentration of 1.5×10^7^ conidia/mL.
• The activity of AChE, α-carboxylesterase, and β-carboxylesterase enzymes were reduced in larvae exposed to *M. anisopliae* conidia.
• *Metarhizium anisopliae* conidia were found to have no significant effect on the terrestrial soil bioindicator earthworm *E. eugeniae*.

## 1 Introduction

According to United Nations forecasts, the global population has increased significantly in recent decades and could reach 9.7 billion people by 2050 (Liu et al., 2017; Fadiji and Babalola, 2020; Bamisile et al., 2021). This expansion necessitates innovative approaches and agricultural applications to secure the sustainability of the food supply (Vos and Bellù, 2019). Accordingly, reducing crop losses is a potential way to increase the quantity of obtainable food materials (Bommarco et al., 2018). The alteration of climatic conditions, outbreaks of various plant-based diseases, and pests are critical factors contributing to the decline of agricultural production globally (Bommarco et al., 2018; Savary et al., 2019). Indeed, the yield of maize, rice, wheat, soybean, potato, barley, cotton, and sugar beet has decreased as a direct result of the impact of pests, which has resulted in an estimated decline of 58% in agricultural production (Oerke, 2006).

Among the agronomy-related pests, the fall armyworm, *Spodoptera frugiperda* (Lepidoptera: Noctuoidea), is an insect pest endemic to both Asia and the Americas. It is a migratory species that feeds on a variety of plant materials and has been found on 353 different host plant species, spanning seventy-six different plant families (Agravante et al., 2022). Furthermore, this pest is capable of infecting cultivated grasses, most notably maize and sorghum (Montezano et al., 2018; Liu et al., 2020; Tambo et al., 2020). *S. frugiperda* is one of the most serious noctuidae pests of maize in Thailand, China, India, and North and South America (Tambo et al., 2020; Castro et al., 2021; Pittarate et al., 2021), owing to its ability to form large populations, the high larval voracity, and high rate of adult dispersal. Recent research reports have shown that *S. frugiperda* is an invasive pest in many countries in Africa and Asia (Fan et al., 2020; Firake and Behere, 2020; Jing et al., 2020; Lee et al., 2020). These countries have favorable climatic conditions and a large variety of plants that can serve as hosts, and, as such, *S. frugiperda* is likely to spread rapidly and cause serious damage to local plants (Wightman, 2018; EFSA, 2020). An estimated $US13 billion in crop losses occur every year in Africa as a result of the fall armyworm. This includes staple crops including corn, rice, and sorghum (Harrison et al., 2019; Tambo et al., 2020; Yigezu and Wakgari, 2020). Therefore, if effective strategies for control are not found or implemented, the pest could pose a serious threat to long-term agricultural productivity and food security.

The search for dependable biological control agents to supplement the use of chemical control began in the early 1980s, when it was discovered that the majority of the insects being managed via chemical control were developing resistance to various types of chemical insecticides and that such chemicals were having a negative impact on beneficial soil living organisms, particularly earthworms (Pelosi et al., 2021). Earthworms are considered a crucial soil indicator species due to their ability to significantly alter the chemical properties of the soil and improve overall soil health (Tondoh et al., 2007; Yuvaraj et al., 2019; Yuvaraj et al., 2020; Yuvaraj et al., 2021a; Yuvaraj et al., 2021b). Due to the widespread use of a variety of insecticides in agricultural soil, earthworm populations have been affected, which has led to a deterioration in the quality of the soil and a subsequent reduction in crop yield (Bagni et al., 2022).

In pursuit of alternative pest control methods to reduce the current overdependence on chemical control, over 750 species of entomopathogenic microorganisms from 85 genera have been found (Liu et al., 2012; Vivekanandhan et al., 2023), including fungi, nematodes, bacteria, and viruses (Shahid et al., 2012; Paschapur et al., 2021). Entomopathogenic fungi can effectively kill a wide range of economically important agricultural insect pests. Bio-insecticides made from entomopathogenic fungi are readily available on the market, including products from the genera *Beauveria* sp., *Cordyceps* sp., *Isaria* sp., *Lecanicillium* sp., *Metarhizium* sp., and *Nomuraea* sp. (Javed et al., 2019; Bara and Laing, 2020; Islam et al., 2021; Rajula et al., 2021; Shehzad et al., 2021; Shehzad et al., 2022; Tu et al., 2023). Because these entomopathogenic fungi are widespread and adaptable, they have been used successfully to eliminate insects in a wide range of habitats around the world (Roberts and Humber, 1981; Scholte et al, 2004; Ambethgar, 2009; Goble et al, 2010; Araújo and Hughes, 2016; Vivekanandhan et al., 2020a). Furthermore, the majority of the entomopathogenic fungi overwinter in the soil, ready to infect the following generation of insect hosts, making biological control an ideal and, eventually, less expensive option than chemical control (Duchowicz et al., 2009; Vivekanandhan et al., 2022a; Vivekanandhan et al., 2022b; Vivekanandhan et al., 2022c).

Specialized enzymes found in insects play vital roles in biochemical and physiological processes (Dittmer and Kanost, 2010). Acetylcholinesterase (AChE), an essential enzyme presents in the nervous system of insects, functions to modulate neurotransmission through the hydrolysis of the neurotransmitter acetylcholine (Colovic et al., 2013). AChE serves a critical role in regulating muscle activity and transmitting nerve signals, making it a target for many insecticides (Colovic et al., 2013; Thapa et al., 2017). Additional enzymes present in insects are β-carboxylesterase and α-carboxylesterase, both of which are involved in metabolic processes and detoxification. These enzymes break down ester compounds as part of a variety of metabolic processes, which also include the detoxification of insecticides and plant defense chemicals. Enzymes thus play a crucial role in insect adaptation by influencing their susceptibility or resistance to pesticides. For this reason, comprehending the biochemistry of these enzymes is imperative in order to formulate efficacious pest management strategies for the benefit of public health and agriculture.

In the current study, the entomopathogenic fungus *Metarhizium anisopliae* was chosen as the study biopesticide because it has been shown to be effective against a wide range of insect pests (Vivekanandhan et al., 2022a) and offers an environmentally friendly and targeted alternative to agricultural pesticides (Bapfubusa Niyibizi et al., 2023). The present investigation aimed to evaluate the physiological and biochemical effects of *M. anisopliae* conidia on the larvae of *S. frugiperda*, as well as the impact on the non-target bioindicator species *Eudrilus eugeniae*.

## 2 Materials and methods

### 2.1 Insect culture

*Spodoptera frugiperda* eggs were procured from a local maize field in Chiang Mai, Thailand (18.7883° N, 98.9853° E). Eggs were placed in a ventilated plastic box (25×15×16 cm) and observed daily until hatching. Neonates (from first–third instars) were maintained on fresh and tender maize leaves in a plastic container. Small plastic boxes (11×8×10 cm) were used for the fourth to fifth instars to avoid cannibalism until pupation. The insect cultures were maintained at a temperature of 27°C and a 12:12 photoperiod (L:D).

### 2.2 *Metarhizium anisopliae* strains

From our previous research, we collected several potent strains of entomopathogenic fungi, particularly *M. anisopliae* (Vivekanandhan et al., 2020a; Vivekanandhan et al., 2020b) (see **Supplementary Figures 1A, B** **).** Potato dextrose agar (PDA) medium was employed to maintain the fungal culture at 27°C for 14 days. After the incubation time, a sterile loop was used to gently scrape the surface of the agar plate to remove conidia (Vivekanandhan et al., 2022a). After aggressively shaking the mixture for three minutes with a 0.05% Tween 80 solution, the hyphae were separated using a sterile millipore cloth. These conidia were spun for twenty minutes to ensure that they were free of clumps. Fungal conidia were counted using a Neubauer hemocytometer chamber, and the active dosage of conidia was estimated using light microscopy (Olympus DSX510i, Japan) at 40X magnification. By diluting the original solution in sterilized distilled water, five distinct concentrations were obtained: 1.5×10^3^, 1.5^5^×10^4^, 1.5×10^5^, 1.5×10^6^, and 1.5×10^7^ conidia/mL. Insecticidal bioassays were executed with these different concentrations.

### 2.3 Effect of *M. anisopliae* against *Spodoptera frugiperda*

Under laboratory conditions, *S. frugiperda* larvae (second-fifth) were tested for larvicidal activity with *M. anisopliae* conidial concentration for up to 9 days. Fresh, immature maize leaves were dipped with various concentrations of fungal conidia (1.5×10^3^, 1.5×10^4^, 1.5×10^5^, 1.5×10^6^, and 1.5×10^7^ conidia/mL), and the treated containers were air dried for 10-15 minutes. Then, twenty-five (second to fifth) instar larvae of *S. frugiperda* were individually transferred into a (5×5×5 cm) plastic bioassay container. In the negative control group, 0.05% Tween-80 was used. Every day, the larvae were fed fresh, surface-sterilized maize leaves. Larval mortality was subsequently assessed for nine days at three-day intervals. Each concentration comprised three replications, and each replication contained twenty-five *S. frugiperda* larval (second-fifth) instars. The treatment bioassay container was incubated at a temperature of 27 ± 2°C.

### 2.4 Light microscopy and colonization assessment

Infected and control larvae were placed in Petri dishes lined with moist filter paper, and incubated at 27 ± 2°C. The fungal colonization of larvae was examined using a light microscope. After 5-7 days of incubation, all larval stages of *S. frugiperda* were evaluated for entomopathogenic fungal mycelium growth.

### 2.5 Effects on haemocyte level

The hemocyte levels of leaf dip treated and control larvae were investigated. Three days post-treatment, hemolymph was collected from the control and experimental groups. The hemolymph was gently inverted two to three times in a 1.5-mL plastic tube with 1 mL of ice-cold anticoagulant buffer (0.01 mL EDTA, 0.1 mL glucose, 0.62 mL NaCl, and 0.26 mL citric acid; pH 4.6) to prevent breakdown. A modified Neubauer hemocytometer (Rohem, India) and a 50-X phase contrast microscope (Magnus MLXi, Japan) were used to count the hemocytes in each corner. The following formula was used to calculate the total hemocyte count: 


$$\text{THC }(\text{Cells}/\text{mm}3)=\frac{X\text{ x dilution factor x depth factor}}{\text{Number of squares counted}}$$

X-Total number of cells counted; Dilution factor-20; Depth factor-10.

### 2.6 Preparation of whole-body homogenates for enzyme assay

Live *S. frugiperda* control and conidia-treated larvae were washed with double-distilled water and wiped with tissue paper to remove excess water. Control and conidia-treated larvae were homogenized in 50 mL of ice-cold sodium phosphate buffer (20 mM, pH 7) to determine enzyme activity levels. The homogenates were previously centrifuged (8000 g for 20 min at 4°C), and the supernatants were used for further investigation. The homogenates were maintained on ice until use in subsequent experiments.

### 2.7 Acetylcholinesterase assay

After larvicidal experiments, control and conidia-treated *S. frugiperda* larvae were separated. Excess water was wiped away before cleaning the larvae. Ellman et al. (1961) employed acetylcholine iodide as a substrate to evaluate the acetylcholine esterase activity of the larval homogenate. 850 mL of 100 mM sodium phosphate buffer was mixed with 50 mL of larval tissues (pH 7.5). Each reaction mixture contained 50 mL of 10 mM DTNB and 50 mL of 12.5 mM cholinergic iodide, which were kept at room temperature for 5 minutes. A suitable blank and a Multiskan EX (200–240 V) spectrophotometer were used to measure the sample’s optical density at 405 nm.

### 2.8 Carboxylesterase assays

We used the method described in Van Asperen (1962) to investigate the activity of α- and β-carboxylesterase enzyme responses in larval homogenate. For the tests, 30 mL of homogenate were combined with 1 mL of sodium phosphate buffer (pH 7.0, 100 mM) containing 250 mM of α- and β-naphthyl acetate. This solution was then maintained at room temperature for 30 minutes. Each reaction mixture was then treated with 400 µL of 0.3% Fast Blue B in 3.3% SDS to block the enzymatic process. The combinations were allowed to remain at room temperature for 15 minutes to develop the distinct color. Multiskan EX-200-240V (Thermo Scientific) and the correct reagent of blank were used to determine the optical density of the sample. The activities of α- and β-carboxylesterase were then determined using a standard blot curve with naphthol as a control.

### 2.9 Earthworm culture

*Eudrilus eugeniae* were maintained at 27 ± 2°C on agricultural waste and cow dung. For experimental purposes, earthworms were removed from the soil by hand sorting. Earthworm bodies were washed with tap water to remove dirt. Earthworm cocoons were counted before being placed on separate bedding.

### 2.10 Artificial soil toxicity analysis

The artificial soil toxicity test was performed in accordance with the method detailed in Vivekanandhan et al. (2022a). The artificial soil was composed of 69% fine sand, 12% crushed sphagnum peat, and 19% kaolinite clay. Small amounts of fine sand, a chemical insecticide (Monocrotophos 250 ppm), and different amounts of fungal conidia were mixed together for each test concentration. After 0.65 kg of dirt and 15 mature earthworms had been added to each 500-mL glass jar, the jars were sealed. The jars were lightly covered with polypropylene covers to allow for ventilation. Jars were kept at a constant temperature of 27 ± 2°C and a relative humidity of 85–90%. Earthworm mortality was assessed after 9 days of treatment.

### 2.11 Histopathological evaluation

Individually, 15 adult *E. eugeniae* were tested for a period of 9 days with either fungal conidia, the organophosphate insecticide Monocrotophos, or distilled water (negative control). Both the control and treated earthworms were fixed in 3% formalin for 3 hours at 4°C after 9 days of treatment. The blocks were cooled for 3 hours at 4°C before being sliced into 2.5-mm-thick ribbons with a microtome (Leica, Germany). Ehrlich’s hematoxylin and eosin were used to stain *E. eugeniae* sections. After staining, the slides were left for drying and later viewed under a light microscope at 40X (Olympus CH20i/India).

### 2.12 Data analysis

The mortality data were subjected to analysis of variance (ANOVA) using logarithmic, arcsine, and square root transformed percentages. The mean of five replications was used to estimate the observed readings. The disparity in the data values among the various treatment groups for larvae was evaluated using Tukey’s multiple range test (p < 0.05) in conjunction with the Minitab®17 software. Sigma Plot 12 (Microcal Software) was employed to calculate the statistical changes for the estimation of mid-gut enzyme. The estimation of the lethal dosage (LC_50_) against larvae after nine days was performed using the Probit study in conjunction with the Minitab®17 software package, with a 95% confidence interval.

## 3 Results

### 3.1 Larvicidal efficacy

**Figure 1:**
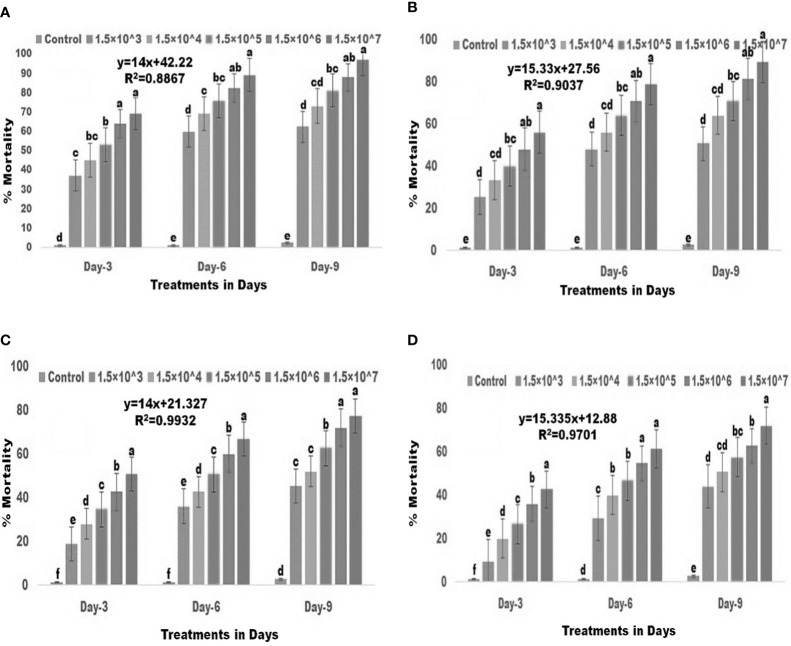
Larvicidal activities of entomopathogenic fungi *M. anisopliae* conidia against 2^nd^ to 5^th^ instar larvae. **(A)** 2^nd^ instar larvae; **(B)** 3^rd^ instar larvae; **(C)** 4^th^ instar larvae; **(D)** 5^th^ instar larvae. Statistical values followed by different letters are significantly different at p≤ 0.05 according to Tukey’s test.

There was a significant difference in larval mortality between the control and conidia-treated groups (**Figures 1A–D**). For second instar larvae, the various fungal conidia concentrations produced 37-69% mortality three days post treatment (df: 5, *F*_(*5,12*)_ =144.071; *p≤* 0.01), 60–89% mortality six days post treatment (df: 5, *F*_(*5,12*)_=264.862; *p≤* 0.01), and 62-97% mortality nine days post treatment (df: 5, *F*_(*5,12*)_=194.480; *p≤* 0.01). When compared to the control (**Figure 1A**), significant differences were observed. For third instar larvae, the fungal conidia produced 25-55% mortality three days post treatment (df: 5, *F*_(*5,12*)_=69.700; *p≤* 0.01), 48-78% mortality six days post treatment (df: 5, *F*_(*5,12*)_=142.322; *p≤* 0.01), and 50-89% mortality nine days post treatment (df: 5, *F*_(*5,12*)_=101.656; *p≤* 0.01) When compared to the control (**Figure 1B**), significant differences were observed. Notable difference in larvicidal activity were observed for forth instar larvae. The fungal conidia showed 18-50% mortality three days post treatment (df: 5, *F*_(*5,12*)_=210.270; *p≤* 0.01), 36-66% mortality six days post treatment (df: 5, *F*_(*5,12*)_=454.450; *p≤* 0.01), and 45-77% mortality nine days post treatment (df: 5, *F*_(*5,12*)_=188.677; *p≤* 0.01). When compared to the control (**Figure 1C**), significant differences were observed. For fifth instar larvae, fungal conidia produced 9-42% mortality three days post treatment (df: 5, *F*_(*5,12*)_=208.200; *p≤* 0.01), 29-61% six days post treatment (df: 5, *F*_(*5,12*)_=195.425; *p≤* 0.01), and 44-72% mortality nine days post treatment (df: 5, *F*_(*5,12*)_=153.492; *p≤* 0.01). When compared to the control (**Figure 1D**), significant differences were observed. Overall, significant differences in larval mortality were observed between control and treatment for all five different concentrations of fungal conidia against second to fifth larval stages of *S. frugiperda*.

### 3.2 Haemocyte level

**Figure 2:**
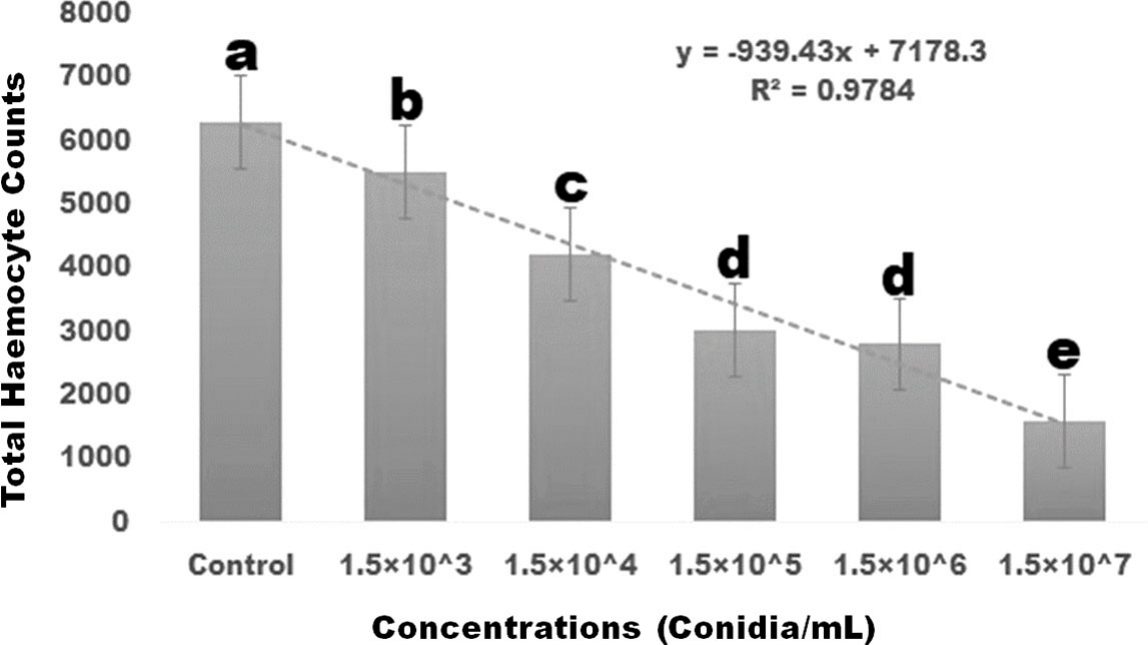
After three days of treatment, the total larval haemocyte counts in *S. frugiperda* larvae decreased in a dose-dependent manner. Statistical values followed by different letters are significantly different at p≤ 0.05 according to Tukey’s test.

*Spodoptera frugiperda* larvae were subjected to *M. anisopliae* fungal conidia at various concentrations (1.5×10^3^, 1.5×10^4^, 1.5×10^5^, 1.5×10^6^, and 1.5×10^7^ conidia/mL). When the concentrations of fungal conidia were increased, haemocyte levels gradually decreased. After three days of treatment, fungal conidia at a higher dose of 1.5×10^7^ conidia/mL reduced haemocyte count by 25.01% and was statistically different from the control (df 5; *F _(5,12)_* = 272.004; *P≤* 0.01) (**Figure 2**).

### 3.3 Acetylcholinesterase assay

**Figure 3:**
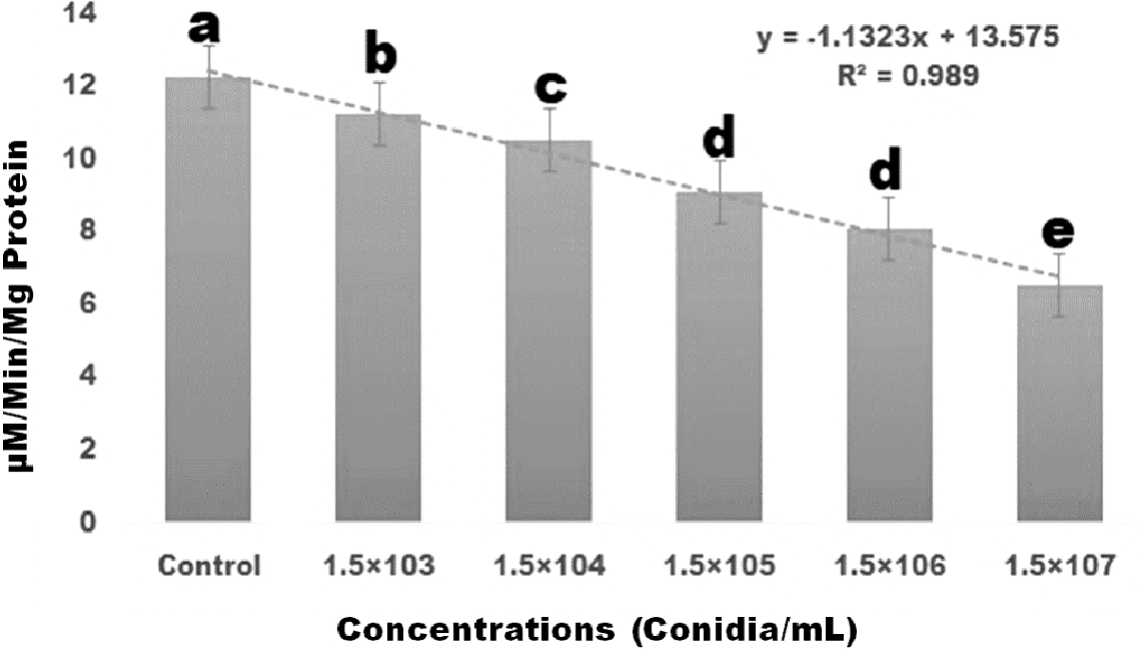
Three days after treatment, *S. frugiperda*’s acetylcholinesterase enzyme response to the insect pathogenic fungus *M. anisopliae* conidia and showed dose-dependent activity. Statistical values followed by different letters are significantly different at p≤ 0.05 according to Tukey’s test.

The biochemical effects of entomopathogenic fungal conidia on *S. frugiperda* larvae were investigated. As conidia concentrations increased, the amount of AchE enzyme activity decreased. A maximal level of AchE was expressed at a concentration of 1.5×10^7^ conidia/mL (df 5; *F *_(*5,12*)_ = 170.162; *p≤* 0.01) (**Figure 3**). The enzyme level in the control larvae was found to be normal. Enzyme expression was mainly dose-dependent, with larvae exposed to entomopathogenic fungal conidia displaying lower levels of AchE enzymes (**Figure 3**).

### 3.4 α -carboxylesterase and β-carboxylesterase assay

**Figure 4:**
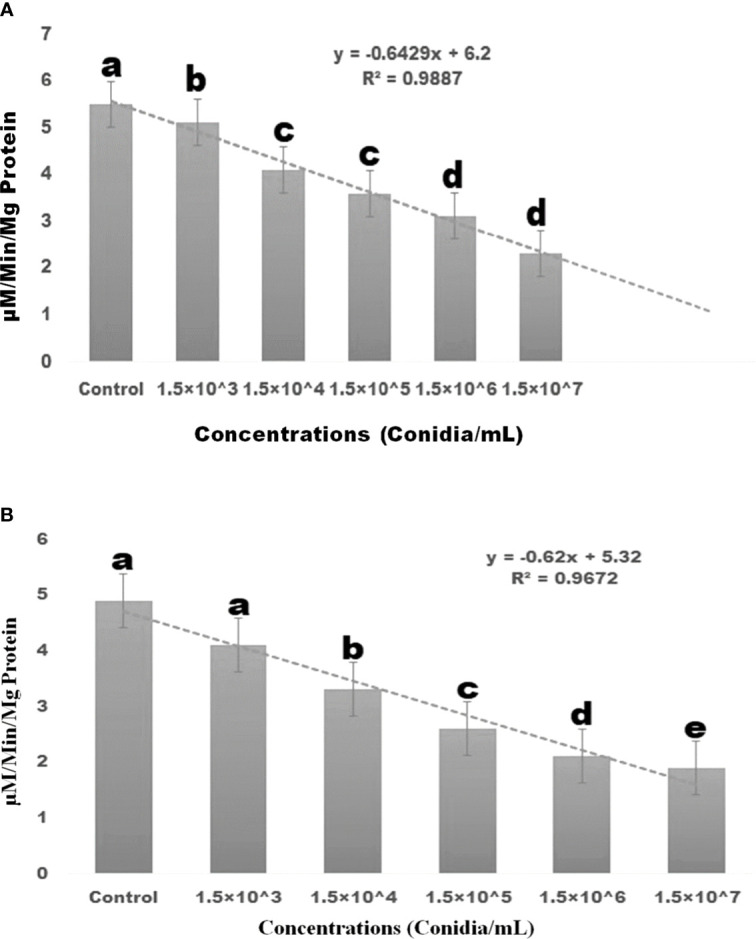
Three days after treatment, *S. frugiperda* α-carboxylesterase and β-carboxylesterase enzyme responses to insect pathogenic fungi *M. anisopliae* conidia caused dose-dependent activity. **(A)** α-carboxylesterase enzyme; **(B)** β-carboxylesterase enzymes. Statistical values followed by different letters are significantly different at p≤ 0.05 according to Tukey’s test.

Results showed that the treatment of *M. anisopliae* fungal conidia against larvae of *S. frugiperda* resulted in a lower level of α and β-carboxylesterase enzyme activity (**Figures 4A, B**), with the lowest level achieved after three days of treatment. *Conidia of M. anisopliae had a dose-dependent effect on the* α and β-carboxylesterase *enzyme activity in larvae.* When larvae were exposed to fungal conidia, their α-carboxylesterase activity was considerably reduced compared to control (5.88 to 2.6 mg protein/mL of homogenate) (df 5; *F*_(*5,12*)_ = 139.597; *p≤* 0.01). (**Figures 4A, B**). A similar finding was observed with β-carboxylesterase, which showed a decrease in activity (from 4.9 to 1.9 mM/protein/mg/min) (df = 5; *F_(5, 12)_* = 172.778; *p≤* 0.01) in *S. frugiperda* larvae (**Figures 4A, B**).

### 3.5 Toxicity on non-target earthworms

**Figure 5:**
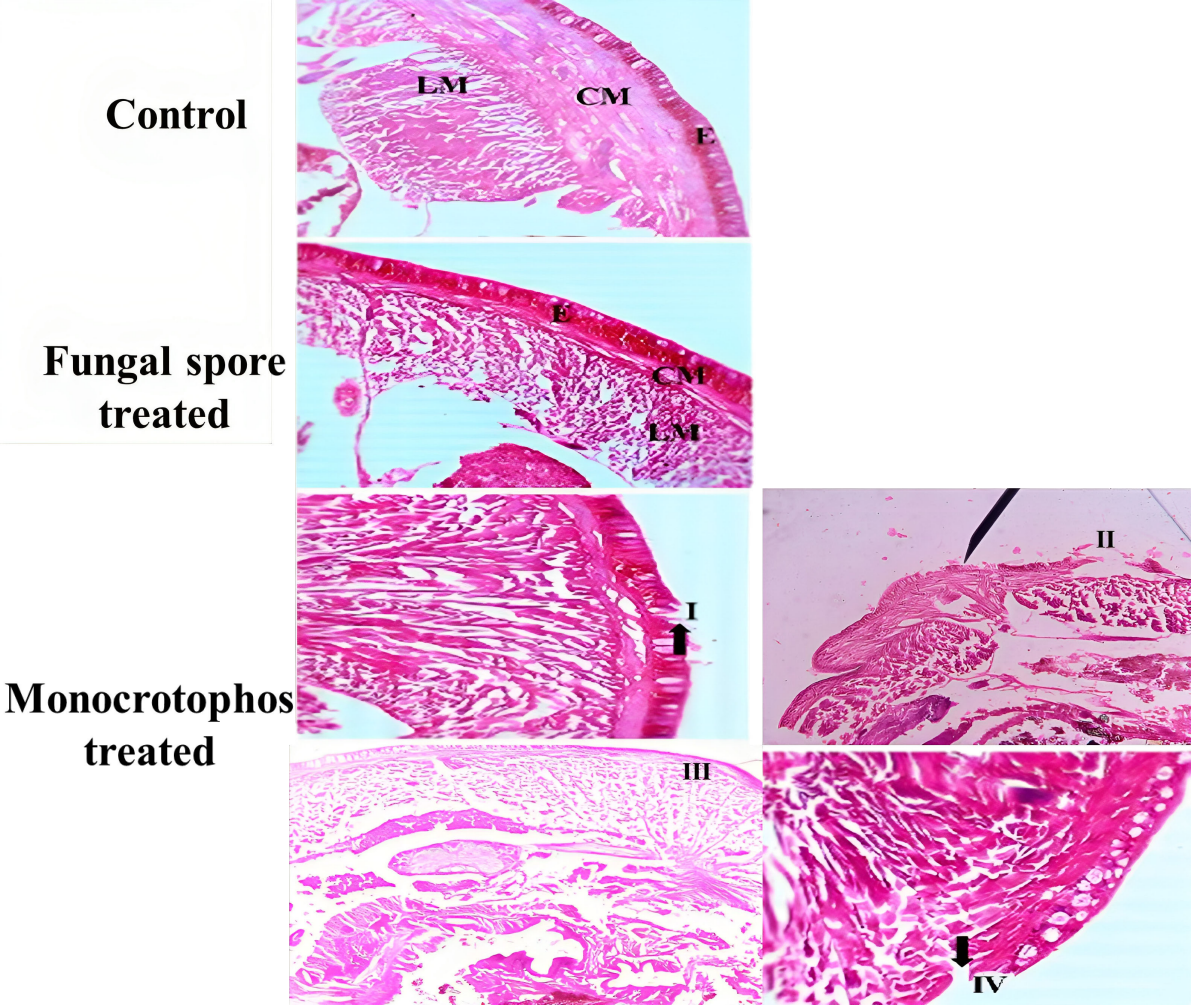
A normal epidermis (E), circular muscle (CM), and longitudinal muscle (LM) can be seen in the cross section of *E. eugeniae*, control, and fungal spore treatments. Monocrotophos treatments resulted in abnormal histology: I, degenerated cells; II, cellular debris; III, an irregular epidermal surface; and IV, degenerated cells.

The results of the artificial soil bioassay revealed that entomopathogenic fungi had no effect on the beneficial non-target species *E. eugeniae*. The results suggests that the mortality rate ranged from 2 to 8% with the treatment of fungal conidia and was not significantly different to control individuals. Fungal conidia thus had no significant effect on earthworm mortality (df = 5; F *_(5, 12)_* = 5.133; *p* 0.01). In contrast, more than 93% of earthworms died because of the monocrotophos treatment. The chemical pesticide, Monocrotophos caused high mortality on *E. eugeniae* when compared to fungal conidia treatment (df = 4; F *_(1, 4)_* = 420.250; *p* 0.01). Monocrotophos was substantially more toxic to the earthworm when compared to *M. anisopliae* conidial concentrations of 1.5×10^7^conidia/mL. Furthermore, *M. anisopliae* fungal conidia were not pathogenic to earthworms after 9 days of treatment. The monocrotophos and control groups were statistically different (df= 4; *F _(1, 4)_* =420.250; *p≤ 0.01).* Monocrotophos was found to be the most dangerous to earthworms when compared to fungal conidia and the negative control (**Figure 5**).

### 3.6 Histopathology evaluation

The histopathological findings revealed that *M. anisopliae* conidia are not toxic to earthworms as compared to Monocrotophos. In the fungal conidia group, treated earthworm gut tissues did not contain any fungal conidia, and the fungal conidia were unable to disintegrate the earthworm epidermis. The fungal conidia had no toxicity effects on earthworm gut tissues; all tissues including the epidermis, circular muscle, setae, mitochondrion, and intestinal lumen tissues were normal and similar to the negative control. In contrast, treatment with monocrotophos resulted in significant damage to the earthworm tissues including the epidermis, circular muscle, setae, mitochondrion, and intestinal lumen tissues (**Figure 5**).

### 3.7 Light microscopy and colonization assessment

*Metarhizium anisopliae* conidia with a green color mycelium completely invaded infected *S. frugiperda* larvae (second to fifth instar). In contrast, control insects were healthy and showed no signs of fungal infection. *M. anisopliae* fungal mycelium has been found to be more capable of proliferating and infiltrating host tissues, resulting in dose-dependent mortality against larvae. Larvae were found to be especially vulnerable to fungal conidia at a concentration of 1.5×10^7^ conidia/mL^-1^.

## 4 Discussion

The current study revealed that *M. anisopliae* fungal conidia caused significant insect larvicidal activity, resulting in up to 97% morality. Among the larval instars of *S. frugiperda*, second and third instars were found to be the most susceptible to *M. anisopliae*. Here, *M. anisopliae* conidia resulted in 97% mortality in the second instar, 87% in the third instar, 77% in the fourth instar, and 72% in the fifth instar larvae at nine days post-treatment. Insect larvae in their early instar stages are more sensitive to fungal infection than larvae in later instars as a result of the immune and physiological systems still developing (Perumal et al., 2023a; Perumal et al., 2023b). Similar to our study, the entomopathogenic fungus, *M. anisopliae* has been previously shown to cause 100% mortality rates in *S. frugiperda* under laboratory trials (Ramos et al., 2020). Furthermore, *M. anisopliae* has successfully suppressed Lepidopteran insects, as evidenced by research on *Plutella xylostella*, which showed the fungi to result in 74% larval mortality (Batta, 2013). It has been previously established that the lethal activity of *M. anisopliae* is proportional to its concentration, with higher concentrations shown to be more effective against insect pests (Opisa et al., 2018). Our findings confirm this, with the maximum mortality being attained with a concentration of 1×10^7^ mL/L. Similar to the present study, *M. anisopliae* 1×10^7^ conidia/mL concentration caused 100% mortality against second instar larvae of *S. exigua* at three days after treatment, and *P. fumosoroseus* caused 100% mortality at six days after treatment using 1×10^4^ conidia/mL (Han et al., 2014). In addition, the current study found that *S. frugiperda* larvae treated with *M. anisopliae* conidia displayed significantly reduced mobility compared to the control group after three days post-treatment. The larvae were observed to be lifeless and had stopped feeding. Many larvae were dead within the first or second day of treatment.

Our results showed that the entomopathogenic fungus *M. anisopliae* conidia reduced larval haemocyte count when compared to the control group after three days post-treatment, with haemocyte count significantly reduced by 25.01% when compared to the control group. Similarly, Karthi et al. (2018); Balumahendhiren et al. (2019) reported that fungal conidial treatment with the entomopathogenic fungi *A. flavus* was capable of significantly reducing the haemocyte count of *Spodoptera litura* larvae at low concentrations (4×10^8^ conidia/ml) within three days. In addition to affecting haemocyte concentration, fungi are capable of influencing the immune system of the pest (Xu et al., 2018; Bai et al., 2022). The biochemical effects of the entomopathogenic fungal conidia on *S. frugiperda* larvae were thus further investigated in this study. Results revealed that, when larvae were exposed to the fungal conidia of *M. anisopliae*, AchE enzyme activity was significantly reduced. This supports previous findings that *A. flavus* fungal conidia treatment reduced AchE enzyme levels in the larvae of *S. litura* under laboratory conditions (Karthi et al., 2018). In addition to impacting AchE enzyme levels, the current study found that the application of *M. anisopliae* conidia to *S. frugiperda* larvae led to a reduction in the levels of α- and β-carboxylesterase enzyme activities. The α-carboxylesterase enzyme levels were reduced from 5.88 to 2.6 mM/protein/mg/min and the β-carboxylesterase enzyme from 4.9 to 1.9 mM/protein/mg/min. The most significant decrease in β-carboxylesterase activity occurred after a treatment period of three days. These results support previously published studies showing that entomopathogenic fungal conidia and secondary metabolites reduce the acetylcholinesterase, α- and β-carboxylesterase enzyme levels in *Spodoptera litura* and *Spodoptera frudiperda* (Karthi et al., 2018; Karthi et al., 2019; Perumal et al., 2023a; Perumal et al., 2023b).

According to the findings of the bioassay conducted on artificial soil, entomopathogenic fungi had no effect on the *E. eugeniae* species, which is a beneficial non-target species. In the current studies, the mortality rate with fungal conidia treatment ranged from 2% to 8%. In contrast, more than 93% of earthworms were dead due to the Monocrotophos treatment. The results demonstrate that fungal conidia have no significant negative impact on earthworms. Similar to the current results, the entomopathogenic fungi *M. anisopliae* was found to have no effect on *E. eugeniae* earthworm species in soil condition (Karthi et al., 2019; Perumal et al., 2023a). Furthermore, *M. majus* conidia did not cause pathogenicity to *E. eugeniae* under laboratory condition (Karthi et al., 2019; Perumal et al., 2023b). Histopathological findings further support that *M. anisopliae* conidia are not hazardous to soil-living earthworm species when compared to chemical pesticides (Monocrotophos). Results showed that *M. anisopliae* conidia do not contain the inside of the earthworm gut tissues. This supports previous research that fungal conidia do not produce pathogenic effects on the earthworm gut tissues (Karthi et al., 2019; Perumal et al., 2023a). Results showed that all earthworm tissues, such as the epidermis, circular muscle, setae, mitochondrion, and intestinal lumen tissues, were normal and similar to the negative control. In contrast, the monocrotophos was completely toxic to earthworm tissues.

In conclusion, results established that entomopathogenic fungal conidia are capable of causing up to 97% mortality rates in *S. frugiperda* larvae, with the second and third instar larvae being the most susceptible to entomopathogenic fungi conidia treatment of the instar stages. Results demonstrated that *M. anisopliae* conidia induce high pathogenicity in the earlier larval stage, and it is therefore expected that the fungi colonized the larval cadaver after the treatment period. In addition, *S. frugiperda* larvae responded to the fungal conidia at three days post-treatment, with haemocyte count significantly reduced when compared to the control group. Results further showed that entomopathogenic fungal conidial infection reduced acetylcholinesterase, α- and β-carboxylesterase enzyme activities in *S. frugiperda* larvae, with enzyme activity reduced in a dose-dependent manner. This study shows that *M. anisopliae* is a potential candidate in the control of *S. frugiperda*.

## Data availability statement

The original contributions presented in the study are included in the article/**Supplementary Material**. Further inquiries can be directed to the corresponding authors.

## Author contributions

PV: Conceptualization, Investigation, Methodology, Project administration, Resources, Supervision, Writing – original draft, Writing – review & editing. KS: Conceptualization, Data curation, Formal Analysis, Resources, Software, Validation, Visualization, Writing – review & editing. AL: Conceptualization, Data curation, Formal Analysis, Software, Validation, Visualization, Methodology, Writing – original draft. PS: Data curation, Formal Analysis, Writing – original draft. KP: Funding acquisition, Investigation, Project administration, Validation, Writing – original draft, Writing – review & editing.
