# Supplementary material for: Entomopathogenic fungi based microbial insecticides and their physiological and biochemical effects on Spodoptera frugiperda (J.E. Smith)
Source: Front Cell Infect Microbiol. 2023 Dec 11;13:1254475. doi: 10.3389/fcimb.2023.1254475 (PMC10750404; doi:10.3389/fcimb.2023.1254475)
Supplement: Supplementary file 1 [file DataSheet_1.docx]

**Entomopathogenic fungi based microbial insecticides and their physiological and biochemical effects on Spodoptera frugiperda** **(J.E. Smith)**

Vivekanandhan Perumal^1^*, Swathy Kannan^1^, Lucy Alford^2^, Sarayut Pittarate^1^, and Krutmuang Patcharin^1^*

^1^Department of Entomology and Plant Pathology, Faculty of Agriculture, Chiang Mai University, Chiang Mai 50200, Thailand.

^2^School of Biological Sciences, Life Sciences Building, University of Bristol, 24 Tyndall Avenue, Bristol BS8 1TQ, United Kingdom.

**Corresponding author*:** [mosqvk@gmail.com (V.P)](about:blank); [patcharin.k@cmu.ac.th](about:blank) (K.P)


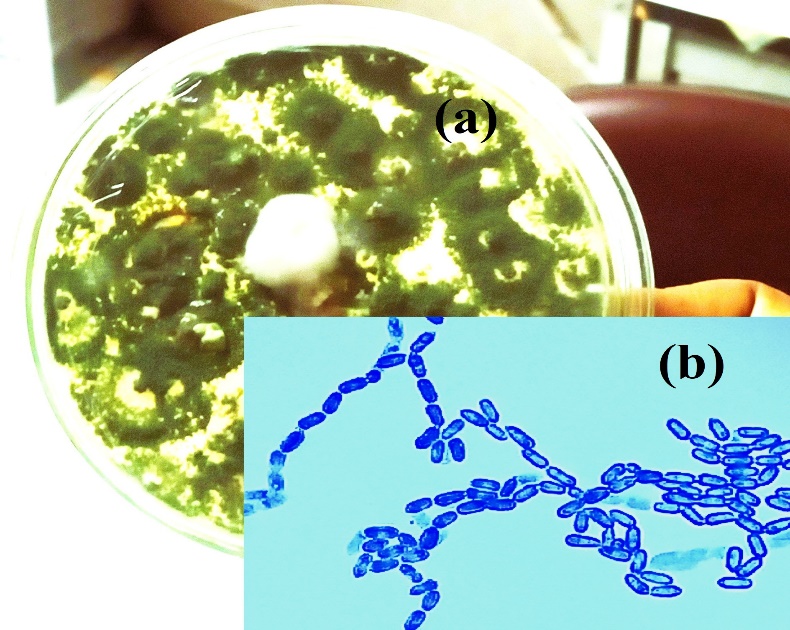


**Figure S1.** The entomopathogenic fungi: A. M. anisopliae fungi; B. Morphological structure of fungal conidia.
